# Pathogenic variants causing *ABL1* malformation syndrome cluster in a myristoyl-binding pocket and increase tyrosine kinase activity

**DOI:** 10.1038/s41431-020-00766-w

**Published:** 2020-11-22

**Authors:** Alexander J. M. Blakes, Emily Gaul, Wayne Lam, Nora Shannon, Karen M. Knapp, Louise S. Bicknell, Meremaihi R. Jackson, Emma M. Wade, Stephen Robertson, Susan M. White, Raoul Heller, Andrew Chase, Diana Baralle, Andrew G. L. Douglas

**Affiliations:** 1 Wessex Clinical Genetics Service, University Hospital Southampton NHS Foundation Trust, Southampton, UK; 2 Human Development and Health, Faculty of Medicine, University of Southampton, Southampton, UK; 3 South East of Scotland Clinical Genetics Service, Western General Hospital, Crewe Road, Edinburgh, UK; 4 Clinical Genetics Service, Nottingham University Hospitals NHS Trust, Hucknall Road, Nottingham, UK; 5 Department of Pathology, Dunedin School of Medicine, University of Otago, Dunedin, New Zealand; 6 Department of Women’s and Children’s Health, Dunedin School of Medicine, University of Otago, Dunedin, New Zealand; 7 Victorian Clinical Genetics Services, Murdoch Children’s Research Institute, Royal Children’s Hospital, Melbourne, VIC Australia; 8 Department of Paediatrics, University of Melbourne, Parkville, VIC Australia; 9 Genetic Health Service NZ - Northern Hub, Auckland District Health Board, Auckland City Hospital, Auckland, New Zealand

**Keywords:** Disease genetics, Congenital heart defects, Musculoskeletal abnormalities, Neurodevelopmental disorders, Genetic testing

## Abstract

*ABL1* is a proto-oncogene encoding a nonreceptor tyrosine kinase, best known in the somatic *BCR-ABL* fusion gene associated with chronic myeloid leukaemia. Recently, germline missense variants in *ABL1* have been found to cause an autosomal dominant developmental syndrome with congenital heart disease, skeletal malformations and characteristic facies. Here, we describe a series of six new unrelated individuals with heterozygous missense variants in *ABL1* (including four novel variants) identified via whole exome sequencing. All the affected individuals in this series recapitulate the phenotype of the *ABL1* developmental syndrome and additionally we affirm that hearing impairment is a common feature of the condition. Four of the variants cluster in the myristoyl-binding pocket of ABL1, a region critical for auto-inhibitory regulation of the kinase domain. Bio-informatic analysis of transcript-wide conservation and germline/somatic variation reveals that this pocket region is subject to high missense constraint and evolutionary conservation. Functional work to investigate ABL1 kinase activity in vitro by transient transfection of HEK293T cells with variant *ABL1* plasmid constructs revealed increased phosphorylation of ABL1-specific substrates compared to wild-type. The increased tyrosine kinase activity was suppressed by imatinib treatment. This case series of six new patients with germline heterozygous *ABL1* missense variants further delineates the phenotypic spectrum of this condition and recognises microcephaly as a common finding. Our analysis supports an ABL1 gain-of-function mechanism due to loss of auto-inhibition, and demonstrates the potential for pharmacological inhibition using imatinib.

## Introduction

*ABL1* is a proto-oncogene encoding a nonreceptor tyrosine kinase with diverse roles in cytoskeleton remodelling and the DNA damage response [1]. It is best known as part of the somatic *BCR-ABL1* fusion gene in the Philadelphia chromosome, associated with chronic myeloid leukaemia (CML) and acute lymphocytic leukaemia (ALL) [2].

*ABL1* spans 170 kb of chromosome 9q34.12, comprising 11 exons. It has two isoforms owing to use of alternative first exons. The longer transcript (NM_007313), encodes 19 additional N-terminal residues involved in auto-inhibition of the ABL1 kinase.

Recently, Wang et al. [3] described an autosomal dominant developmental syndrome (MIM 617602) caused by germline heterozygous missense variants (NM_007313.2:c.734A > G p.(Tyr245Cys) and c.1066G > A p.(Ala356Thr)) in *ABL1*. Clinical features included congenital heart disease, skeletal malformations, dysmorphic facies, and failure to thrive. More recently, the clinical spectrum of the ABL1 malformation syndrome has been expanded to include hearing impairment, renal hypoplasia and ocular abnormalities [4].

Tyr245 lies in the SH2-kinase linker domain of ABL1 essential for the “docking” of the Src homology (SH3) domain in the inactive conformation of the protein [5]. Docking of SH3 to the SH2-kinase linker domain is one of three “linchpins” proposed to hold ABL1 in an inactive closed state, and therefore has an important auto-inhibitory role [6]. Phosphorylation of Tyr245 is necessary for maximal wild-type ABL1 kinase activity; a p.Tyr245Phe substitution reduces ABL1 kinase activity by 50% in vitro [7]. Unexpectedly, Wang et al. found that the c.734A > G p.(Tyr245Cys) variant increased ABL1 kinase activity [3], suggesting a possible gain-of-function effect.

Ala356 lies within the myristoyl-binding pocket of the ABL1 kinase domain. In isoform 1b (NM_007313) a myristoyl group bound to the N-terminal glycine of ABL1 occupies this pocket to stabilise the inactive conformation of the protein [8]. This docking of the myristoyl residue is the second “linchpin” of ABL1 auto-inhibition, which is lost in the BCR-ABL fusion product as the ABL1 N-terminus is truncated. The substitution of Ala356 for the polar amino acid threonine is expected to disrupt important hydrophobic interactions within the pocket. Indeed, the c.1066G > A p.(Ala356Thr) variant has increased kinase activity in vitro [3], consistent with a gain of function due to failure of auto-inhibition.

The developmental significance of ABL1 is illustrated by animal models. *Abl*^*2*^ mice harbour a targeted insertion-deletion in which exon 5 and part of exon 6 are replaced by the neomycin resistance gene [9]. Homozygotes die soon after birth with thymic and splenic atrophy, lymphopenia and osteoporosis [10]. *Abl*^*m1*^ mice have a large targeted deletion of approximately one third of the ABL1 protein from the c-terminus [11]. Homozygotes have increased perinatal mortality, with defects of spleen, head and eye development [12]. Interestingly, *Abl*^*m1*^ homozygotes of a C57BL/6J background also develop cardiac abnormalities [13]. Heterozygotes of both strains are largely unaffected, suggesting that *ABL1* does not display haploinsufficiency, and supporting the possibility that the human germline missense variants act through a gain of function.

In this study, we have collected clinical and molecular details of six patients with deleterious *ABL1* variants and have modelled the effects of these variants in vitro. We find that all but one of the variants identified in this cohort cluster in the myristoyl-binding pocket of ABL1, and that these variants increase the tyrosine kinase activity of ABL1 in vitro. These results are consistent with a gain-of-function effect, in which the variants disrupt the crucial auto-inhibitory binding of an N-terminal myristoyl group to its binding pocket. We find that variants in this myristoyl-binding pocket are a common cause of the *ABL1* cardiac and skeletal malformation syndrome.

## Subjects and methods

### Patients

Patients 1, 3 and 5 were identified through genetic variant results returned via the Deciphering Developmental Disorders (DDD) study [14] (Complementary Analysis Project #278; DECIPHER IDs: patient 1 = 304716, patient 3 = 300146, patient 5 = 304918). UK ethical approval for the DDD study has been granted by the Cambridge South Research Ethics Committee (10/H0305/83). Patients 2, 4 and 6 were identified as part of routine clinical practice through clinical genetics services in Australia and New Zealand. Informed consent for publication was obtained for all patients whose clinical details and clinical photographs are included in this report. Ethical approval for the study involving patient 4 was obtained from the New Zealand Health and Disability Ethics Committee (16/STH/3). Clinicians of all patients reported to have *ABL1* variants were contacted and requested to make assessments of variant pathogenicity in their patients.

### Genetic analysis

For patients 1, 3 and 5, whole exome sequencing of saliva DNA samples was carried out through the DDD study. The DDD sequencing and bioinformatics framework has been previously described [15]. The DDD study identified 23 patients with missense variants in *ABL1*. Variants deemed pathogenic or likely pathogenic among those patients included in this report were confirmed by Sanger sequencing. The exome sequencing strategies used to identify the variants in patients 2 and 4 have been previously described [16, 17]. Each variant was confirmed by Sanger sequencing. Patient 6 underwent whole exome sequencing through Invitae (boosted exome, proband only). Genomic DNA was enriched using a proprietary hybridisation-based protocol and sequenced on an Illumina platform. Sequences were aligned to GRCh37. Mean sequencing depth was 230x, with 99.9% of positions in reportable exons covered at >20x. Minimum calling depth was at least 20x. Targeted regions included at least 95% of the mappable exome, ±10 bp flanking regions. Promoters, untranslated and other non-coding regions were not interrogated. Variants were identified using a proprietary calling algorithm and confirmed by Sanger sequencing. Variants are annotated against GenBank transcript ID NM_007313.2. Exons are numbered as for GenBank accession NG_012034.1. The variants identified in this study have been submitted to the ClinVar database (accession numbers SCV001441170–SCV001441174).

### Plasmid mutagenesis

A pCDNA3.1/V5-His A plasmid vector containing the *ABL1* cDNA sequence was gifted by Yaping Yang, Baylor College of Medicine (Houston, TX, USA). Plasmid mutagenesis of the ABL1B transcript (NM_007313.2) was carried out for each variant following a modified version of the QuikChange Site-Directed Mutagenesis method (Agilent Technologies, Manchester, UK) using PfuUltra II Fusion HotStart DNA Polymerase (see Supplementary Information for details). Primers were designed using Agilent’s QuikChange Primer Design online tool (https://www.chem.agilent.com/store/primerDesignProgram.jsp) or using a partially overlapping primer design [18] (see Supplementary Information for primer sequences). Mutagenised plasmids were used to transform One Shot TOP10 Chemically Competent *E. coli* (Thermo Fisher Scientific, Paisley, UK). Individual clones were isolated and *ABL1* fully sequenced to confirm the correct sequence and presence of the required variant.

### Transfection and ABL1 activity assay

To investigate ABL1 kinase activity in vitro, HEK293T cells were transfected with plasmid constructs encoding wild-type or variant *ABL1* cDNA using Lipofectamine 2000 (Thermo Fisher Scientific, Paisley, UK). After 48 h, cells were serum starved for 1 h before preparation of protein lysates. Phosphorylation of ABL1 and the ABL1-specific substrate STAT5B was measured by western blotting using the following antibodies: ABL1 (clone OP20) (EMD Millipore, Billerica, MA, USA); Phospho-ABL1 (Tyr245) (Cell Signaling Technology, Danvers, MA, USA, #2861); Phospho-ABL1 (Tyr241) (Abcam, Cambridge, UK), STAT5 (Cell Signaling Technology, #9363); phospho-STAT5 (Cell Signaling Technology, #9359); Phosphotyrosine (Fisher Scientific, PY20), Actin (Santa Cruz Biotechnology, Dallas, Texas, USA; SC-10731). Imatinib was purchased from Stratech (Ely, UK). To assess effect on phosphorylation activity, 1 µM imatinib was added during the 1 h serum starvation before lysis.

### Analysis of variation and conservation in *ABL1*

Non-pathogenic missense variants of *ABL1* in patients with abnormal phenotypes were collated from the DECIPHER database [14]. *ABL1* missense variants in healthy population controls were identified through the gnomAD [19] and EVS [20] databases. PhyloP basewise evolutionary conservation scores [21] for every position in the transcript (NM_007313.2) were obtained through the UCSC table browser [22]. Missense constraint scores for every codon were obtained from the MTR Gene Viewer [23]. Pathogenic variants were analysed by the standard pathogenicity prediction programs PolyPhen [24], SIFT [25], MutationTaster [26], and CADD [27].

## Results

### Clinical features

**Fig. 1:**
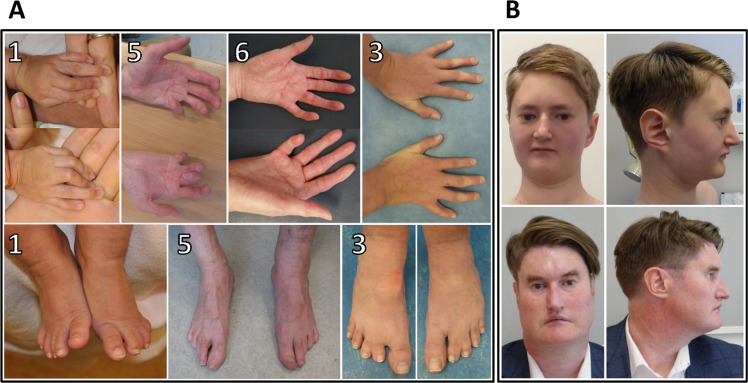
Clinical photographs of patients with the *ABL1* cardiac and skeletal malformation syndrome. **a** Hands and feet of patients 1, 3, 5 and 6. Features include camptodactyly (patients 1, 3, 5 & 6), 5th finger clinodactyly (patients 3 and 6), bilateral Duputyen’s contracture (patient 5), and 2–3 toe syndactyly (patient 3). **b** Facial photographs of patients 3 (top) and 6 (bottom). Note the small chin, down-turned mouth, and long, narrow nose.

The clinical features of our cohort are summarised in Table 1, and representative clinical photographs are given in Fig. 1. All six individuals recapitulate the phenotype of congenital heart disease, skeletal malformations and characteristic facies which had been previously described. Hearing impairment has recently been identified as a common feature of the ABL1 malformation syndrome [4]; four of our cohort exhibit conductive or mixed conductive/sensorineural hearing impairment, which was severe and persistent in one patient. Interestingly, two individuals have tall stature, in contrast to short stature in the majority of cases. Some other phenotypic features are also over-represented in our cohort, including camptodactyly (5/6) and microcephaly (5/6). Others are under-represented or absent, including pectus deformity (1/6), ear abnormalities (1/6), gastro-intestinal disorders (1/6), joint hyper-extensibility (0/6), dental decay (0/6), and genito-urinary disorders (1/6) (Table S1).

**Table 1:** Clinical details of patients with *ABL1* missense variants.

General	Patient	1	2	3	4	5	6
	Variant	c.1066G>A; p.(Ala356Thr)	c.1066G>A; p.(Ala356Thr)	c.1354G>A; p.(Ala452Thr)	c.1574T>C; p.(Val525Ala)	c.1582G>A; p.(Glu528Lys)	c.731T>C; p.(Val244Ala)
General	Age (years)	4	29	13	6	40	37
	Gender	Female	Female	Male	Female	Male	Male
Growth	Age at measurement	3.5 years	25 years	13 years	6 years	40 years	36 years
	Height/Length (cm)	92.4 (<9th centile)	150.8 (<0.4th centile)	170.9 (98th centile)	109 (9th centile)	191 (<99.6th centile)	188 (98th centile)
	Weight (kg)	11.6 (0.4th centile)	37.6	56.3 (91st centile)	(9th-25th centile)	82.5	103
	Head circumference (cm)	46 (<0.4th centile)	51 (<3rd centile)	52.1 (2nd centile)	45 (<2nd centile)	52.6 (<<0.4th centile)	60.5 (90th centile)
	Intrauterine growth restriction	Yes (BW 2.2kg at 37 weeks)	Yes (BW 2.41kg at 36 weeks)	No (BW 2.75kg at 37 weeks)	Yes (mild)	No (BW 3.09kg at 41 weeks)	Yes
	Growth failure	Yes	Yes	Yes	Yes	Yes	Yes
	Feeding difficulties					Yes	Yes
	Stature	Short stature	Short stature	Tall stature	Short stature (mild)	Tall stature (proportionate)	Tall stature
Development	Developmental delay		No	Moderate (global)	Mild	Mild	Mild (mainly motor)
Dysmorphic features	Face	High-arched eyebrows, full cheeks	Elongated face, narrow maxilla, facial asymmetry, scaphocephaly			High-arched eyebrows	Broad forehead, narrow maxilla, long face
	Eyes		Deep-set eyes	Almond-shaped eyes, epiblepharon	Epicanthic folds	Ptosis, proptosis	
	Ears			Asymmetry of the ears		Prominent ears, lobeless ears	
	Nose	Hypoplastic alae nasi	Long narrow nose, hypoplastic alae nasi	Prominent nasal tip	Narrow overhanging nasal tip, broad nasal root	Prominent nasal bridge, low columella	Long narrow nose
	Mouth		Thin lips, dental crowding	Small down-turned mouth			Down-turned mouth
	Palate		High-arched palate			High palate	
	Chin	Microretrognathia	Pointed chin	Small pointed chin	Small pointed chin	Micrognathia	Pointed chin
Cardiovascular	Atrial spetal defect	No	No	Yes	Yes	Yes	No
	Ventricular septal defect	No	Yes	Yes	No	Yes	No
	Aortic root dilatation	No	Yes (mild)	Yes (mild)	No	Yes	Yes (mild)
	Other		Supra-valvular pulmonary stenosis	Patent ductus arteriosus		Bicuspid aortic valve, pacemaker for intermittent junctional rhythm	Idiopathic hypertension, mild concentric left ventricular hypertrophy
Skeletal	Pectus excavatum			Yes			
	Scoliosis		Yes (surgical intervention)			Yes (thoracic)	
	Finger/toe abnormality	Camptodactyly of fingers, arachnodactyly	Camptodactyly of fingers	2–3 toe syndactyly, camptodactyly of fingers, clinodactyly of 5th fingers	2–3 toe syndactyly, clinodactyly of 4th and 5th fingers, tapered fingers	Camptodactyly, bilateral Dupuytren’s contracture, slender fingers	Camptodactyly, clinodactyly of 5th finger, slender fingers
	Foot deformity			Metatarsus adductus			Mild hindfoot valgus deformity
	Other			Hypoplasia of right lower limb			
Joints	Hypermobility	None	Mild elbow laxity	Joint laxity (Beighton score 4/9)	None		None
	Other					Joint swelling of fingers, osteoarthritis of hips	
Gastrointestinal	Constipation/Reflux		No	Constipation	No		No
Genito-urinary	Renal tract		No	Left renal agenesis	No		
	Reproductive tract			Absent left vas deferens			Micropenis, hydrocoele
Skin	Thin skin		Yes		Yes		Yes
	Other		Fibromata of hands and feet				Cutis marmorata
Other	Hearing impairment	Congenital conductive hearing impairment	Chronic otitis media, secondary conductive hearing loss		Mixed conductive/sensorineural hearing impairment (50dB loss bilaterally)	Mixed conductive/sensorineural hearing impairment	
	Other		Lacrimal duct stenosis, recurrent pneumothorax	Bilateral inguinal hernias, fetal choroid plexus cysts, spontaneous pneumothorax		Varicose veins, liver cirrhosis	Oligodontia, distal upper limb weakness, prominent veins

Adult head circumference centiles are based on charts produced by Bushby et al [34].

*BW* birth weight.

The most common clinical features across all described individuals with *ABL1* variants are dysmorphic facies (18/18), finger/toe abnormalities (17/18), congenital heart disease (14/18), failure to thrive (14/18), developmental delay (11/18), IUGR (10/18), ear abnormalities (9/18), palatal deformity (9/18) and microcephaly (9/18) (Table S1).

### Pathogenic *ABL1* variants

**Fig. 2:**
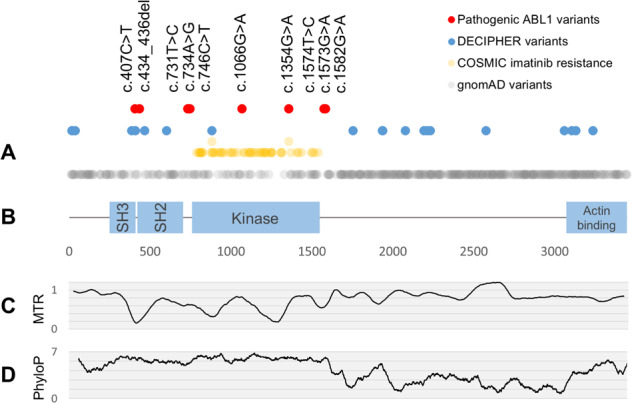
Pathogenic and benign variation, missense constraint and evolutionary conservation in *ABL1* (NM_007313.2). **a** Pathogenic and benign variants in *ABL1*. Red points indicate pathogenic germline *ABL1* variants described here and previously. Blue points indicate non-pathogenic *ABL1* missense variants in DECIPHER. Yellow points indicate somatic missense variants in haematological malignancy associated with Tyrosine Kinase Inhibitor (TKI) resistance in the COSMIC database. Raised yellow points indicate that this variant is also seen in the germline in a DECIPHER participant. Grey points indicate missense variants in gnomAD. **b** Schematic of functional domains in *ABL1*, with amino acid residue labelled on the horizontal axis. Pathogenic missense variants cluster near the kinase domain of the transcript, as do somatic missense variants conferring resistance to imatinib. The kinase domain is also depleted for non-pathogenic variants in DECIPHER, and for benign variation in gnomAD. **c** Missense constraint in *ABL1*. Moving average of Missense Tolerance Ratio (MTR) scores with 20 codon window. MTR scores represent the missense tolerance of *ABL1* codons, derived from the prevalence of missense variation in the ExAC cohort. Lower scores indicate codons which are under missense constraint. Codons in the kinase domain and SH3/2 domains are under greater missense constraint than the remainder of the transcript. **d** Basewise conservation in *ABL1*. Moving average of basewise PhyloP scores with 60 base window. Higher scores indicate more highly conserved bases. Bases at the 5′ end of the transcript, comprising the SH3, SH2, and kinase domains, tend to be more highly conserved than the remainder of the transcript.

**Fig. 3:**
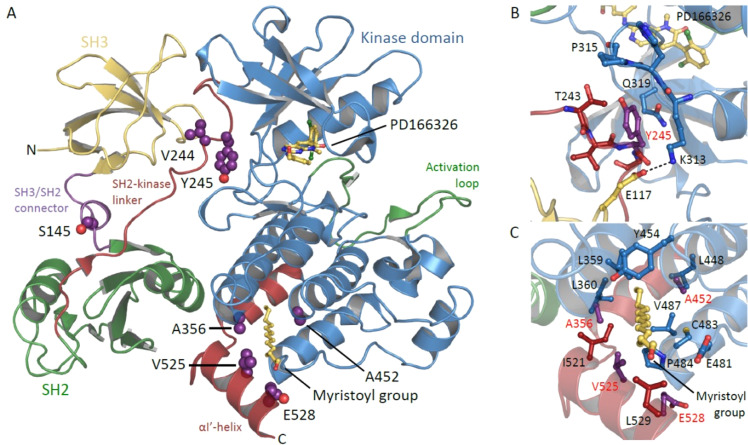
Structure of autoinhibited ABL1 showing locations of patient missense variants. **a** Cartoon representation of autoinhibited ABL1 in complex with ATP-competitive inhibitor PD166326 and the myristoyl group of a myristoylated peptide (both shown in stick representation), with side-chains of patient missense variants shown as purple spheres (PDB: 1OPL). **b** Closeup view showing that the Tyr245 side-chain packs in a hydrophobic crevice formed by the side-chains of Lys313 and Pro315 of the kinase domain. Substitution with a cysteine would abolish phosphorylation at this site and may disrupt an important salt bridge between Lys313 and Glu117. **c** Closeup view showing that Ala356, Ala452, Val525 and Glu528 cluster at the myristoyl-binding pocket and form important hydrophobic interactions with the myristoyl group and to other amino acids that complete the binding pocket. Amino acid substitutions observed in patients at these sites are likely to impact binding of the myristoylated peptide.

We identified six individuals with five deleterious de novo missense variants in *ABL1*. Four of these variants have not been previously described. Our findings are summarised in Fig. 2. Four of the five variants cluster in the myristoyl-binding pocket within the kinase domain, which is a critical auto-inhibitory region in ABL1 (Fig. 3).

The molecular characteristics of each variant are shown in Table 2. The nucleotide and amino acid at each position is highly conserved between species. All variants are predicted to be deleterious by multiple pathogenicity prediction programs. None of the variants are present in gnomAD. All novel variants were classified as “pathogenic” or “likely pathogenic” by ACMG criteria [28].

**Table 2:** Molecular details of deleterious *ABL1* missense variants.

General	Patient	1	2	3	4	5	6
Molecular labels	Position (hg19/GRCh37)	9:133748348	9:133748348	9:133753828	9:133755890	9:133755898	9:133738274
	Exon Number	6	6	8	10	10	4
	Transcript (RefSeq)	NM_007313.2	NM_007313.2	NM_007313.2	NM_007313.2	NM_007313.2	NM_007313.2
	c.	c.1066G > A	c.1066G > A	c.1354G > A	c.1574T > C	c.1582G > A	c.731T > C
	p.	p.(Ala356Thr)	p.(Ala356Thr)	p.(Ala452Thr)	p.(Val525Ala)	p.(Glu528Lys)	p.(Val244Ala)
Conservation	Nucleotide (phyloP)	Highly conserved 6.067	Highly conserved 6.067	Highly conserved 4.161	Highly conserved 4.998	Highly conserved 6.049	Highly conserved 4.736
	Amino Acid Conservation	*D. melanogaster*	*D. melanogaster*	*C. elegans*	*X. tropicalis*	*D. rerio*	*F. catus*
Pathogenicity	ExAC Allele Frequency	0	0	0	0	0	0
	Missense Tolerance Ratio	0.846	0.846	0.657	0.584	0.583	0.6
	SIFT	Damaging 0.019	Damaging 0.019	Damaging 0.013	Damaging 0.007	Damaging 0.048	Damaging 0.02
	Mutation Taster	Disease causing P: 0.999	Disease causing P: 0.999	Disease causing P: 0.999	Disease causing P: 0.999	Disease causing P: 0.999	Disease causing P: 0.998
	CADD	31	31	27	28.5	33	27.3
	ACMG Classification	Pathogenic	Pathogenic	Likely Pathogenic	Likely pathogenic	Likely pathogenic	Uncertain^a^
Inheritance	Inheritance	De novo	De novo	De novo	De novo	De novo	Unknown
	Zygosity	Heterozygous	Heterozygous	Heterozygous	Heterozygous	Heterozygous	Heterozygous

^a^The variant in patient 6 would be classified as “Likely pathogenic” if PP4 were applied (highly specific phenotype for a disease with a single genetic aetiology) or “Pathogenic” if PS3 were applied in light of the experimental findings in this paper (functional studies supportive of a damaging effect).

### Benign *ABL1* variation

Whereas pathogenic heterozygous germline *ABL1* variants cluster within and adjacent to the *ABL1* kinase domain, non-pathogenic *ABL1* variants in the DECIPHER cohort largely lie outside this region (Fig. 2a). Benign germline variation among gnomAD participants is found in every domain of *ABL1* but is relatively scarce within the kinase domain (Fig. 2a). Mean PhyloP scores are significantly higher in the kinase domain and the SH3/2 domains than the rest of the transcript, while MTR scores are correspondingly lower in these regions (Fig. 2b, Supplementary Table 2). Codons and individual bases within the kinase domain are therefore prone to greater missense constraint and evolutionary conservation than other positions in the transcript.

Somatic *ABL1* missense variants associated with imatinib resistance in BCR-ABL leukaemias cluster exclusively within the kinase domain (Fig. 2a). One residue (Ala452) is associated both with pathogenic variation in the germline, and imatinib resistance as a somatic variant.

### In vitro ABL1 assay

**Fig. 4:**
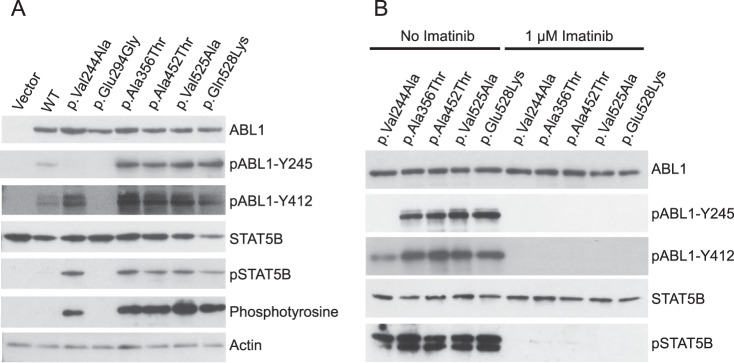
Missense variants cause increased ABL1 tyrosine kinase activity in vitro. **a** Tyrosine kinase activity of ABL1 missense constructs. Missense variants NM_007313.2:c.1066G > A p.(Ala356Thr), c.1354G > A p.(Ala452Thr), c.1574T > C p.(Val525Ala), and c.1582G > A p.(Glu528Lys) markedly increase the phosphorylation of ABL1 at residue Tyr245, and the phosphorylation of the ABL1-specific substrate STAT5B, compared to wild-type. The c.881A > G p.(Glu294Gly) construct (for which the variant is not thought to be deleterious), does not increase phosphorylation of ABL1 or STAT5B. The c.731T > C p.(Val244Ala) construct increased phosphorylation of STAT5B and tyrosine phosphorylation overall, but not at ABL1-Tyr245. Substitution of Val244 for alanine therefore appears to result in loss of phosphorylation of the adjacent Tyr245 residue. These findings are consistent with gain of ABL1 tyrosine kinase activity due to loss of auto-inhibition through myristoyl binding. **b** Treatment with 1 µM imatinib results in complete loss of phosphorylation activity.

To investigate ABL1 kinase activity in vitro, HEK293T cells were transfected with plasmid constructs encoding wild-type or variant *ABL1* cDNA. Cell lysates were assayed for phosphorylation of ABL1-specific substrates by immunoblotting (Fig. 4). Phosphorylation of ABL1-Tyr245 and STAT5B were substantially increased in lysates transfected with the c.1066G > A p.(Ala356Thr) construct, consistent with previous reports [3]. Phosphorylated ABL1 and STAT5B were also increased for the c.1354G > A p.(Ala452Thr), c.1574T > C p.(Val525Ala), and c.1582G > A p.(Glu528Lys) constructs (Fig. 4a). These results are consistent with gain of ABL1 tyrosine kinase activity due to loss of auto-inhibition by myristoyl binding.

No evidence of ABL1 activation was seen for the negative control, c.881A > G p.(Glu294Gly). This variant was identified through the DDD study in a patient with a likely pathogenic de novo variant in another gene.

The c.731T > C p.(Val244Ala) construct caused an increase in phosphorylation of STAT5B and of overall tyrosine phosphorylation, with reduced autophosphorylation at Tyr245 compared to wild-type. Alteration of Val244 to alanine therefore appears to result in loss of phosphorylation of the adjacent Tyr245 residue. This result is surprising, as phosphorylation of Tyr245 is thought to potentiate ABL1 kinase activity [7]. However, the previously described pathogenic c.734A > G p.(Tyr245Cys) variant also abolishes phosphorylation at this site [3]. Furthermore, other variants within the SH2-catalytic domain linker region have previously been shown to cause ABL1 to adopt an active conformation by disrupting the inhibitory interaction between the SH3 and catalytic domains [7, 29].

Finally, as expected, imatinib abolished phosphorylation of ABL1-Tyr245 and STAT5B in all of the constructs (Fig. 4b).

## Discussion

We describe six additional patients with a skeletal and cardiac malformation syndrome caused by heterozygous missense variants in *ABL1*, which in five cases are confirmed as de novo. All but one of these variants cluster in a myristoyl-binding pocket within the kinase domain, suggesting a gain-of-function effect due to loss of auto-inhibition. We present functional data consistent with this mechanism and reaffirm that the clinical phenotype of the ABL1 syndrome includes conductive hearing loss.

A distinctive feature of the variants we describe is their close spatial relationship to one another in the three-dimensional crystal structure of the protein, and specifically their position within the myristoyl-binding pocket. This close spatial relationship is not immediately apparent from the position of these variants in the *ABL1* transcript, and yet suggests a self-evident mechanism by which they can exert a deleterious gain-of-function effect. We predict that other amino-acid substitutions within the myristoyl-binding pocket, particularly those which disrupt hydrophobic interactions or introduce bulky amino acids, will also be deleterious in the germline. We also predict that missense variation or in-frame deletion of the N-terminal glycine of ABL1, which carries the myristoyl modification, will be deleterious.

We also describe a novel c.731T > C p.(Val244Ala) variant which lies in the SH2-kinase linker domain, immediately adjacent to a previously described c.734A > G p.(Tyr245Cys) variant. Other variants in this linker domain are known to disrupt the inhibitory docking of the SH3 domain to the SH2-kinase linker domain, and thereby constitutively activate the ABL1 kinase [7]. Notably, the c.731T > C p.(Val244Ala) variant we describe causes reduced phosphorylation of ABL1-Tyr245 in vitro. Phosphorylation of Tyr245 is necessary for maximal activation of the wild-type ABL1 [7], yet the c.731T > C p.(Val244Ala) and c.734A > G p.(Tyr245Cys) variants must activate ABL1 independently of the Tyr245 phosphorylation status. Recently, an in-frame deletion of c.434_436del p.(Ser145del) has also been associated with the *ABL1* developmental syndrome [30], but no functional work has yet been performed to characterise the effect of this variant on ABL1 kinase activity.

*ABL1* is best known as a proto-oncogene. In CML and other haematological malignancies, a somatic translocation between chromosomes 9 and 22 produces the Philadelphia chromosome [31], carrying a *BCR-ABL* fusion gene. As the N-terminus of ABL1 is lost in the fusion product, the auto-inhibitory binding of the myristoyl group to the kinase domain is abolished, and ABL1 gains constitutive tyrosine kinase activity which drives cellular proliferation [32].

Tyrosine kinase inhibitors (TKIs) specific to ABL1, such as imatinib, are the mainstay of treatment for CML. However, resistance to TKI therapy is strongly associated with somatic missense variants in the ABL1 kinase domain, particularly in the ATP-binding loop (P loop) and at TKI-specific binding sites [33].

It is noteworthy that none of the activating germline variants we describe have been associated with somatic TKI resistance. If, as we argue, germline variants in the kinase domain sterically hinder myristoyl binding, they will functionally mimic the loss of the ABL1 N-terminus in BCR-ABL. We therefore expect that TKIs effective against BCR-ABL should be similarly effective against these variant proteins. Indeed, TKIs may potentially in some way be therapeutically beneficial for the *ABL1* developmental syndrome or its complications, for example in limiting aortic root dilatation or reducing the tendency for dilatation to occur. However, given that this condition appears to affect embryonic and fetal development, any more complete therapeutic effect would require as early treatment as possible and entail long-term therapy, potentially risking adverse drug effects. While imatinib is used to treat paediatric CML cases, it is expected to be teratogenic in pregnancy. Further work is therefore required to ascertain whether therapeutic scope exists for use of imatinib or similar TKIs in this condition.

It is also noteworthy that activating somatic missense variants in *ABL1* have not been found to independently cause haematological malignancy, although both isoforms of *ABL1* are ubiquitously expressed. Disruption of myristoyl binding alone may not activate ABL1 sufficiently to drive malignancy. Two other “linchpins” of ABL1 auto-inhibtion (the docking of the SH3 domain to a polyproline helix in the SH2-kinase linker, and an N-terminal “brace” over the SH3-SH2 unit) may prevent its excessive activation [6]. Indeed, both the N-terminal “brace” and the myristoyl group are lost in the BCR-ABL fusion. It is not clear whether the activating germline variants we describe can act as driver variants in malignancy. No patients with the *ABL1* skeletal and cardiac malformation syndrome described here or elsewhere are reported to have haematological malignancy, but longitudinal follow up of these patients will be required to better determine this.

From a clinical perspective, we believe this condition to be a phenotypically distinctive and recognisable syndrome based on affected individuals’ dysmorphology and associated clinical features. Phenotypes such as skeletal malformations, aortic root dilatation and pneumothorax point towards an overlap with genetic connective tissue disorders and we recommend that *ABL1* be borne in mind in such cases. The high prevalence of hearing impairment makes audiological assessment advisable. Furthermore, assessment of the aortic root diameter at the time of diagnosis may also be appropriate in individuals found to have pathogenic *ABL1* variants. Evidence is currently lacking as to the true risk of aortic aneurysm and dissection in this condition. We are not aware of any affected individuals having had rapid progressive aortic dilatation requiring surgical intervention and this may therefore suggest a more indolent course. However, a precautionary approach of ongoing aortic root screening similar to that used for Marfan syndrome may be appropriate until such time as more accurate natural history data are available.

## Supplementary information

Supplementary information

## References

[1] Colicelli J (2010). ABL tyrosine kinases: evolution of function, regulation, and specificity. Sci Signal.

[2] de Klein A, van Kessel AG, Grosveld G, Bartram CR, Hagemeijer A, Bootsma D (1982). A cellular oncogene is translocated to the Philadelphia chromosome in chronic myelocytic leukaemia. Nature..

[3] Wang X, Charng WL, Chen CA, Rosenfeld JA, Al Shamsi A, Al-Gazali L (2017). Germline mutations in ABL1 cause an autosomal dominant syndrome characterized by congenital heart defects and skeletal malformations. Nat Genet.

[4] Chen C, Crutcher E, Gill H, Nelson TN, Robak LA, Jongmans MCJ (2020). The expanding clinical phenotype of germline ABL1 ‐associated congenital heart defects and skeletal malformations syndrome. Hum Mutat..

[5] Hantschel O, Superti-Furga G (2004). Regulation of the c-Abl and Bcr-Abl tyrosine kinases. Nat Rev Mol Cell Biol.

[6] Nagar B, Hantschel O, Seeliger M, Davies JM, Weis WI, Superti-Furga G (2006). Organization of the SH3-SH2 unit in active and inactive forms of the c-Abl tyrosine kinase. Mol Cell.

[7] Brasher BB, Van Etten RA (2000). c-Abl Has High Intrinsic Tyrosine Kinase Activity That Is Stimulated by Mutation of the Src Homology 3 Domain and by Autophosphorylation at Two Distinct Regulatory Tyrosines. J Biol Chem.

[8] Hantschel O, Nagar B, Guettler S, Kretzschmar J, Dorey K, Kuriyan J (2003). A myristoyl/phosphotyrosine switch regulates c-Abl. Cell.

[9] Tybulewicz VLJ, Crawford CE, Jackson PK, Bronson RT, Mulligan RC (1991). Neonatal lethality and lymphopenia in mice with a homozygous disruption of the c-abl proto-oncogene. Cell.

[10] Li B, Boast S, De Los Santos K, Schieren I, Quiroz M, Teitelbaum SL (2000). Mice deficient in Abl are osteoporotic and have defects in osteoblast maturation. Nat Genet.

[11] Schwartzberg PL, Goff SP, Robertson EJ (1989). Germ-line transmission of a c-abl mutation produced by targeted gene disruption in ES cells. Science..

[12] Schwartzberg PL, Stall AM, Hardin JD, Bowdish KS, Humaran T, Boast S (1991). Mice homozygous for the ablm1mutation show poor viability and depletion of selected B and T cell populations. Cell..

[13] Qiu Z, Cang Y, Goff SP (2010). c-Abl tyrosine kinase regulates cardiac growth and development. Proc Natl Acad Sci.

[14] Firth HV, Richards SM, Bevan AP, Clayton S, Corpas M, Rajan D (2009). DECIPHER: database of Chromosomal Imbalance and Phenotype in Humans Using Ensembl Resources. Am J Hum Genet.

[15] Wright CF, Fitzgerald TW, Jones WD, Clayton S, McRae JF, Van Kogelenberg M (2015). Genetic diagnosis of developmental disorders in the DDD study: a scalable analysis of genome-wide research data. Lancet..

[16] Wade EM, Daniel PB, Jenkins ZA, McInerney-Leo A, Leo P, Morgan T (2016). Mutations in MAP3K7 that Alter the Activity of the TAK1 Signaling Complex Cause Frontometaphyseal Dysplasia. Am J Hum Genet.

[17] Knapp KM, Poke G, Jenkins D, Truter W, Bicknell LS (2019). Expanding the phenotypic spectrum associated with DPF2: a new case report. Am J Med Genet Part A.

[18] Zheng L, Baumann U, Reymond J-L (2004). An efficient one-step site-directed and site-saturation mutagenesis protocol. Nucleic Acids Res.

[19] Lek M, Karczewski KJ, Minikel EV, Samocha KE, Banks E, Fennell T (2016). Analysis of protein-coding genetic variation in 60,706 humans. Nature..

[20] Exome Variant Server. NHLBI GO Exome Sequencing Project (ESP), Seattle, WA. http://evs.gs.washington.edu/EVS/. Accessed December 2018.

[21] Pollard KS, Hubisz MJ, Rosenbloom KR, Siepel A (2010). Detection of nonneutral substitution rates on mammalian phylogenies. Genome Res.

[22] Karolchik D, Hinrichs AS, Furey T, Roskin KM, Sugnet CW, Haussler D (2004). The UCSC Table Browser data retrieval tool. Nucleic Acids Res.

[23] Traynelis J, Silk M, Wang Q, Berkovic SF, Liu L, Ascher DB (2017). Optimizing genomic medicine in epilepsy through a gene-customized approach to missense variant interpretation. Genome Res.

[24] Adzhubei I, Jordan DM, Sunyaev SR (2013). Predicting functional effect of human missense mutations using PolyPhen-2. Curr Protoc Hum Genet.

[25] Kumar P, Henikoff S, Ng PC (2009). Predicting the effects of coding non-synonymous variants on protein function using the SIFT algorithm. Nat Protoc.

[26] Schwarz JM, Cooper DN, Schuelke M, Seelow D (2014). MutationTaster2: mutation prediction for the deep-sequencing age. Nat Methods.

[27] Rentzsch P, Witten D, Cooper GM, Shendure J, Kircher M (2019). CADD: predicting the deleteriousness of variants throughout the human genome. Nucleic Acids Res.

[28] Richards S, Aziz N, Bale S, Bick D, Das S, Gastier-Foster J (2015). Standards and guidelines for the interpretation of sequence variants: a joint consensus recommendation of the American College of Medical Genetics and Genomics and the Association for Molecular Pathology. Genet Med.

[29] Barilá D, Superti-Furga G (1998). An intramolecular SH3-domain interaction regulates c-Abl activity. Nat Genet.

[30] Bravo-Gil N, Marcos I, González-Meneses A, Antiñolo G, Borrego S (2019). Expanding the clinical and mutational spectrum of germline ABL1 mutations-associated syndrome. Med (Baltim).

[31] Nowell P, Hungerford D (1960). A minute chromosome in human chronic granulocytic leukemia. Science..

[32] Nagar B, Hantschel O, Young MA, Scheffzek K, Veach D, Bornmann W (2003). Structural basis for the autoinhibition of c-Abl tyrosine kinase. Cell..

[33] Chahardouli B, Zaker F, Mousavi SA, Saffari Z, Nadali F, Ostadali M (2013). Detection of BCR-ABL kinase domain mutations in patients with chronic myeloid leukemia on imatinib. Hematology..

[34] Bushby KMD, Cole T, Matthews JNS, Goodship JA (1992). Centiles for adult head circumference. Arch Dis Child.

