# Supplementary material for: Pathogenic variants causing ABL1 malformation syndrome cluster in a myristoyl-binding pocket and increase tyrosine kinase activity
Source: Eur J Hum Genet. 2020 Nov 22;29(4):593–603. doi: 10.1038/s41431-020-00766-w (PMC8115115; doi:10.1038/s41431-020-00766-w)
Supplement: Supplementary file 1 — Supplementary information [file 41431_2020_766_MOESM1_ESM.docx]

# **Supplementary information**

## **Table S1. Combined phenotype information**

##
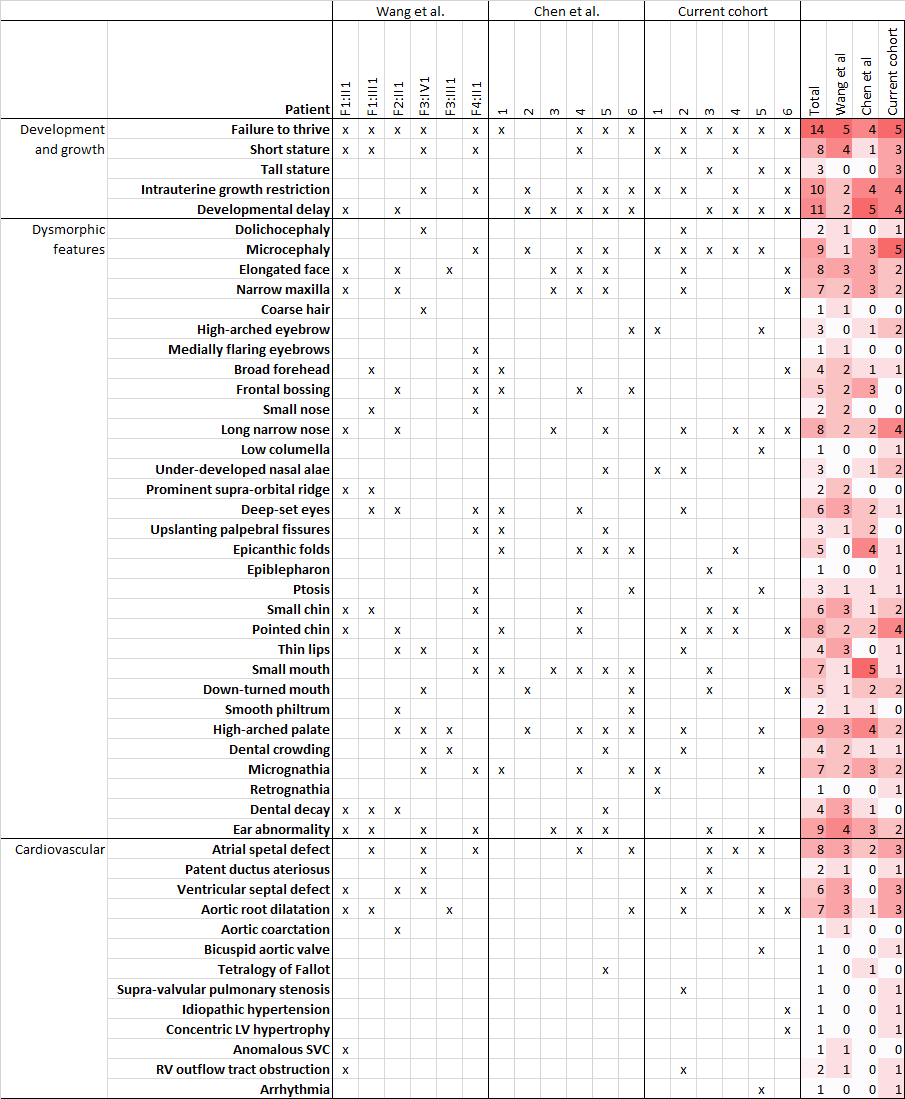


## **Table S1. Combined phenotype information (continued)**


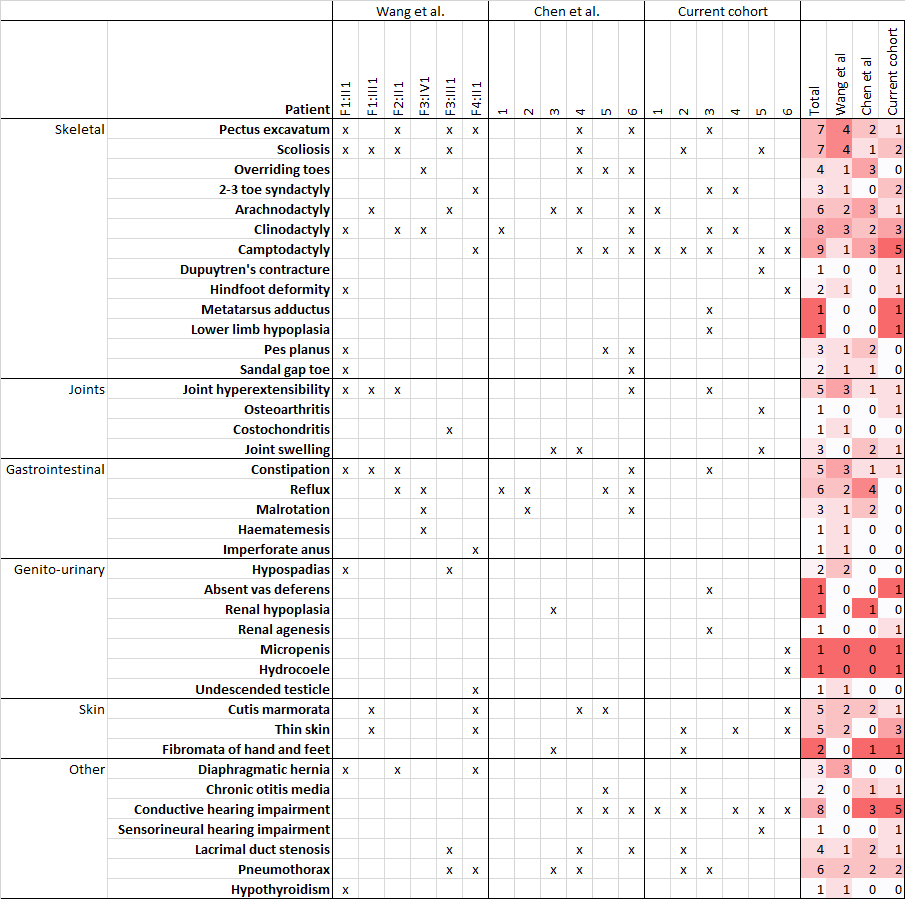


## **Table S2**.

Mean scores of missense tolerance and conservation in *ABL1* domains. P-values calculated by comparison of MTR or phyloP scores in *ABL1* domains against all positions in *ABL1* which lie outside of these domains. The non-parametric Wilcoxon rank sum test (one sample, unpaired) was used as scores are not normally distributed.

## **Clinical summaries**

## **Patient 1 (DECIPHER 304716) – *ABL1* NM_007313.2:c.1066G>A p.(Ala356Thr)**

This patient is an 18-month-old girl, who in pregnancy was found to have intrauterine growth retardation and was born at 37 weeks gestation weighing 2.2 kg. She had poor weight gain and at assessment had a length and head circumference on the 0.4^th^ centile with weight below the 0.4^th^ centile. She has arachnodactyly of the hands with fixed flexion deformity of the proximal interphalangeal joint bilaterally, which is improving. Additionally, she has bilateral conductive hearing loss. Her developmental milestones have been appropriate for her age. She has fine eyebrows, full cheeks, hypoplastic alae nasi and micrognathia. Through trio whole-exome sequencing she was found to have a *de novo* *ABL1* NM_007313.2:c.1066G>A p.(Ala356Thr) variant, assessed as "pathogenic" by ACMG criteria.

## **Patient 2 – *ABL1*** **NM_007313.2:c.1066G>A p.(Ala356Thr)**

This patient is a 25-year-old female from a non-consanguinous family of Anglo-Celtic origin with no family history of a similar presentation. She was born at 36/40 gestation, birth weight 2.41kg (3rd centile). In infancy a ventricular septal defect was diagnosed requiring surgical repair aged one year. She had short stature (height on the 3rd centile) and microcephaly (head circumference <1st centile) but normal cognitive development. She has chronic conductive hearing loss initially treated with tympanostomy tubes, resulting in chronic perforation of the right tympanic membrane. In her teenage years mild aortic root dilatation was detected on echocardiogram in childhood but her most recent echocardiogram showed normal aortic dimensions. She has developed a progressive scoliosis requiring surgical intervention and also progressive camptodactyly of the hands, associated with purplish discolouration over the proximal and distal interphalangeal joints. Biopsy of this tissue demonstrated that these lesions were fibromata. Similar lesions have developed on the sole of her foot during the third decade. She has a history of recurrent pneumothoraces in adolescence. Her facial features show scaphocephaly, marked facial asymmetry, a long narrow face with a pinched nose and hypoplastic alae nasi. She successfully completed a Bachelor of Science degree at university. Investigations have included a normal spine MRI with no dural ectasia, a chromosomal microarray and sequencing of *TGFBR1* and *TGFBR2* including deletion/duplication analysis which demonstrated no pathogenic variants. Karyotypic analysis of dermal fibroblasts demonstrated no abnormalities. Using a trio exome sequencing approach she was found to have a *de novo* *ABL1* NM_007313.2:c.1066G>A p.(Ala356Thr) variant, assessed as class 5 "pathogenic" using ACMG criteria.

## **Patient 3 (DECIPHER 300146) – *ABL1* NM_007313.2:c.1354G>A p.(Ala452Thr)**

This patient is a 13-year-old boy, the first child of unrelated parents. He was born at 37 weeks gestation weighing 2.75 kg. Prenatally there had been choroid plexus cysts and unusual positioning of the hands. Amniocentesis confirmed a normal male karyotype. After birth he was found to have an atrial septal defect, ventricular septal defect and a patent ductus arteriosus, for which he underwent corrective surgery during infancy. He is not known to have any aortic root dilatation. His development was mildly delayed, walking at around 18 months and requiring speech and language therapy up until the age of three years. He attends mainstream school but with support. He required bilateral inguinal hernia repair aged two years and was also found to have an absent left kidney and vas deferens. He has mild myopia but has also required surgical correction of bilateral epiblepharon. He has metatarsus adductus of his right foot and a degree of right lower limb hypoplasia, as well as bilateral cutaneous 2-3 toe syndactyly. X-rays revealed an unusual wedge-shaped configuration of the middle phalanges of his second toes. At the age of 12 he developed a spontaneous right-sided pneumothorax, requiring thoracoscopic bullectomy and pleurodesis. Facially he has a small pointed chin, small down-turned mouth, almond-shaped eyes, and asymmetric flattening of his right pinna. He has tall stature (98^th^ centile) with relative microcephaly (2^nd^ centile) and has an arm span greater than his height but with a span-to-height ratio below 1.05. He has short fifth fingers with clinodactyly and has camptodactyly affecting his proximal interphalangeal joints. He has pectus excavatum and an elongated ribcage and has a degree of hypermobility with a Beighton score of 4/9. He was found to have a *de novo ABL1* NM_007313.2:c.1354G>A p.(Ala452Thr) variant, assessed as class 4 “likely pathogenic” using ACMG criteria^1^.

## **Patient 4 – *ABL1* NM_007313.2:c.1574T>C p.(Val525Ala)**

This patient is a 6-year-old female with no family history of a similar presentation. She was born after a pregnancy complicated by mild intrauterine growth retardation and postnatally by failure to thrive and feeding problems necessitating placement of a percutaneous feeding tube. Her stature has tracked at -1.1 SD but her head circumference has been persistently small with it measuring -4.6 SD at 6 years of age. Her neurodevelopment is mildly delayed with relative strengths in sociability and weaknesses in expressive language and coordination of gross motor tasks. Her clinical presentation is notable for craniofacial dysmorphism (deep-set eyes, a broad nasal base and a small, pointed chin), thin translucent skin, mixed sensorineural/conductive deafness, an atrial septal defect and near complete cutaneous syndactyly of toes 2/3. Investigations demonstrated a normal serum 7-dehydrocholesterol level, no imbalance on chromosomal microarray, a normal renal ultrasound and EEG. An MRI showed normal brain structure. Using a trio exome sequencing approach she was found to have a *de novo* *ABL1* NM_007313.2:c.1574T>C p.(Val525Ala) variant, assessed as class 4 "likely pathogenic" using ACMG criteria.

## **Patient 5 (DECIPHER 304918) – *ABL1* NM_007313.2:c.1582G>A p.(Glu528Lys)**

This gentleman is the third child to non-consanguineous parents. He was small for dates on scan but was born by normal vaginal delivery at term with a birth weight of 3.09 kg (15th centile). He was found to have an ASD, VSD and bicuspid aortic valve at 3 weeks of age. He was treated with pulmonary artery banding at 3 months of age and then closure of his ASD and VSD at 3 years of age. He remained well from a cardiovascular point of view until he was investigated for syncopal episodes at 19 yrs of age and had a dual chamber pacemaker inserted for an intermittent junctional rhythm. This was subsequently removed after he developed endocarditis and septic shock requiring ECMO support. He was found to have a moderate hearing loss at the age of 5 years which was associated with ear infections initially but persisted as a mixed conductive/sensorineural loss. He has a narrow left external auditory canal. He had a bilateral congenital ptosis which was surgically corrected in 2006. There were feeding difficulties in infancy and he could only manage small regular feeds. His weight gain was slow and he was always tall for his age. He attended a school for children with learning difficulties until the age of 11 years when his time was divided between mainstream school and a special school environment. He went on to attend a catering college and work independently. When he was first seen at the age of 26 years he had limited movement at the distal interphalangeal joints and slight swelling of the proximal interphalangeal joints in his hands. He also had camptodactyly of his toes. Over a period of 15 years he has experienced an increase in the pain, swelling and flexion contractures in his fingers and has also developed bilateral Dupytren contracture. In addition, he has had pains in his hip joints and lower back and x rays confirmed early osteoarthritic changes in his hips. Aortic root dilatation has been present from the age of 33 years. At the age of 35 yrs he developed extensive varicose veins in his legs which caused problems with bleeding and were treated surgically. He developed liver cirrhosis with portal hypertension and gastric varices at the age of 40 yrs. On examination he has a height of 191cm (98th centile), weight of 82.5 kg (93rd centile) and head circumference of 52.6cm (0.4th centile). He has a tall, slim body habitus with a normal span to height ratio of 0.98. He had proptosis, ptosis, arched eyebrows, micrognathia, a prominent nasal bridge and low columella. His palate is high with no dental crowding and a normal uvula. He has lobeless, prominent helices. He has no pectus and a mid-thoracic scoliosis. He has long slim fingers and toes with marked fixed camptodactyly and swelling of his distal and proximal interphalangeal joints. He also has camptodactyly of his third to fifth toes and prominent varicose veins over his lower legs. His skin has become more translucent with age, especially over his lower limbs. He has a *de novo* variant in *ABL1* NM_007313.2:c.1582G>A, p.(Glu528Lys) classified as "likely pathogenic" according to ACMG criteria.

## **Patient 6 – *ABL1* NM_007313.2:c.731T>C p.(Val244Ala)**

This patient is a 37-year-old male professional with an unremarkable family history. He was born at term after a pregnancy complicated by intrauterine growth restriction from 20 weeks’ gestation. He exhibited mildly delayed gross motor development, congenital weakness of the distal muscles of the upper limbs, and congenital camptodactyly of fingers and toes which responded to physiotherapy. He has some facial dysmorphism with a long face, narrow maxilla, prominent nose, broad forehead, and macrocephaly. He had cytogenetic investigation in childhood and was found to have a normal 46,XY karyotype on G-banding. Since childhood he has had prominent veins and thin skin on the back of his hands. He has no skin fragility, unusual bleeding, or impaired wound healing. He has oligodontia; all permanent canines are absent. He has no other ectodermal symptoms such as brittle or sparse hair or nails, or anhydrosis. He has had unexplained hypertension since age 36. An echocardiogram revealed normal heart structures, mild concentric LV hypertrophy. A subsequent cardiac MRI showed minimal dilatation of the aortic root and ascending aorta and significant left ventricular systolic dysfunction with an ejection fraction of 46%. Exome sequencing identified an *ABL1* NM_007313.2:c.731T>C p.(Val244Ala) variant which was confirmed by Sanger sequencing.

**Supplementary methods**

**Site-directed mutagenesis PCR**

## Plasmid constructs of pCDNA3.1/V5-His A incorporating wild-type *ABL1B* sequence underwent 18 cycles of PCR with a 60°C annealing temperature and 68°C extension step of 7 minutes using the following reagents:

| **Reagent** | **Volume (μl)** |
| --- | --- |
| 10x PfuUltra II reaction buffer | 5 |
| dNTPs (10 mM) | 2.5 |
| Q solution | 3 |
| Forward primer (10 **μM)** | 1 |
| Reverse primer (10 **μM)** | 1 |
| PfuUltra II fusion HS DNA polymerase | 1 |
| Nuclease-free water | 35.5 |
| Plasmid template DNA (50-100 ng) | 1 |

Methylated template DNA was digested by adding 1 **μl** *DpnI* (10 U/**μl) for 2 hours at 37**°C prior to bacterial transformation.

## **Mutagenesis primer sequences**

| Variant (NM_007313.2) | Forward mutagenesis primer | Reverse mutagenesis primer |
| --- | --- | --- |
| c.1066G>A p.(Ala356Thr) | GCAGGAGGTGAACACCGTGGTGCTGCT | AGCAGCACCACGGTGTTCACCTCCTGC |
| c.1582G>A p.(Glu528Lys) | GGAAAAGAAGCTGGGGAAACAAGGCGTCCG | CCCAGCTTCTTTTCCACTTCGTCTGAGATAC |
| c.1574T>C p.(Val525Ala) | GACGAAGCGGAAAAGGAGCTGGGGAAACAAG | CCTTTTCCGCTTCGTCTGAGATACTGGATTCC |
| c.731T>C p.(Val244Ala) | GCAACAAGCCCACTGCCTATGGT | TTGGGGGACACACCATAGGCAGTGG |
| c.1354G>A p.(Gln528Lys) | GCATTTGGAGTATTGCTTTGGGAAATTACTACCTATGGCATGT | ACATGCCATAGGTAGTAATTTCCCAAAGCAATACTCCAAATGC |
| c.881A>G p.(Glu294Gly) | GTGAAGACCTTGAAGGGGGACACCATGGAGGTG | CACCTCCATGGTGTCCCCCTTCAAGGTCTTCAC |

**References**:

1 Richards S, Aziz N, Bale S *et al.* Standards and guidelines for the interpretation of sequence variants: a joint consensus recommendation of the American College of Medical Genetics and Genomics and the Association for Molecular Pathology. *Genet Med* 2015; **17**: 405–423.
